# Producing Vaccines against Enveloped Viruses in Plants: Making the Impossible, Difficult

**DOI:** 10.3390/vaccines9070780

**Published:** 2021-07-13

**Authors:** Hadrien Peyret, John F. C. Steele, Jae-Wan Jung, Eva C. Thuenemann, Yulia Meshcheriakova, George P. Lomonossoff

**Affiliations:** Department of Biochemistry and Metabolism, John Innes Centre, Norwich NR4 7UH, UK; hadrien.peyret@jic.ac.uk (H.P.); jfcsteele@gmail.com (J.F.C.S.); Jae-Wan.Jung@jic.ac.uk (J.-W.J.); eva.thuenemann@jic.ac.uk (E.C.T.); Yulia.Meshcheriakova@jic.ac.uk (Y.M.)

**Keywords:** alphavirus, *Bunyavirales*, coronavirus, *Flaviviridae*, hepatitis B virus, human immunodeficiency virus, Influenza virus, newcastle disease virus, plant molecular farming, plant-produced vaccines, rhabdovirus, virus-like particles

## Abstract

The past 30 years have seen the growth of plant molecular farming as an approach to the production of recombinant proteins for pharmaceutical and biotechnological uses. Much of this effort has focused on producing vaccine candidates against viral diseases, including those caused by enveloped viruses. These represent a particular challenge given the difficulties associated with expressing and purifying membrane-bound proteins and achieving correct assembly. Despite this, there have been notable successes both from a biochemical and a clinical perspective, with a number of clinical trials showing great promise. This review will explore the history and current status of plant-produced vaccine candidates against enveloped viruses to date, with a particular focus on virus-like particles (VLPs), which mimic authentic virus structures but do not contain infectious genetic material.

## 1. Introduction

As a consequence of their size and geometry, self-assembling macromolecules, such as virus-like particles (VLPs), have proven to be one of the most effective ways of eliciting an immune response in recipients [1,2,3]. Thus, many recombinant vaccines and vaccine candidates are based on VLPs. These can consist either of a common scaffold on which antigenic sequences are displayed or, in the case of viruses, non-infectious VLPs of the parental virus itself. In either case, the VLP can be expressed recombinantly in several different expression systems including *E. coli*, yeast, insect cells, mammalian cells and plants. This review will focus on efforts to produce vaccine candidates against enveloped viruses in plants, with a particular focus on VLPs.

Plants have the theoretical advantages that high yields and low costs are potentially possible, they are attractive for the development of vaccines that could be administered orally, and they are very unlikely to be contaminated with an adventitious mammalian pathogen. There has therefore been considerable interest in expressing VLPs in plants as a means of producing vaccines for veterinary and medical use. Considerable success has been achieved in displaying antigens or producing VLPs from non-enveloped viruses [4,5], including the stimulation of protective immunity in both experimental and target animals [6,7,8]. There are, however, fewer successful demonstrations of immunogenicity in the case of enveloped viruses, especially in the case of VLP production as opposed to antigen display. This undoubtedly reflects the greater complexity of the particles of enveloped viruses as they contain both host- and virus-derived components and the proteins undergo significant post-translational modifications. Here we review the progress that has been made to date and assess the prospects for the future.

## 2. Approaches

There have been essentially two approaches to the creation of VLPs of enveloped viruses in plants. The first involves identifying the virus-derived proteins that are known to self-assemble inside infected cells and expressing those proteins either through stable transgenesis or transiently. If the envelope proteins are expressed, the production of VLPs relies on the ability of these proteins to interact productively with plant membranes. Fortunately, this has frequently been found to be the case [9,10,11].

The alternative approach is to use a preformed scaffold to display antigenic sequences from enveloped viruses to create virus-specific VLPs [12]. Prominent among such scaffolds are particles from plant viruses, including those with both isometric and helical morphologies. These include cowpea mosaic virus (CPMV), tobacco mosaic virus (TMV), potato virus X (PVX), tomato bushy stunt virus (TBSV) and cucumber mosaic virus (CMV). Details of the deployment of these different viruses, and the sequences from enveloped viruses that have been expressed on their surfaces can be found in previous reviews [13]. However, plant virus particles are not the only self-assembling macromolecules that have been used as scaffolds for antigen display in plants. Particles from hepatitis B virus (HBV) core antigen (HBcAg) produced in plants have been used (numerous examples are discussed below) as well as non-viral molecules, such as flagellin, which has been used to present the M2e epitope from influenza virus [14,15].

## 3. HBV

**Figure 1:**
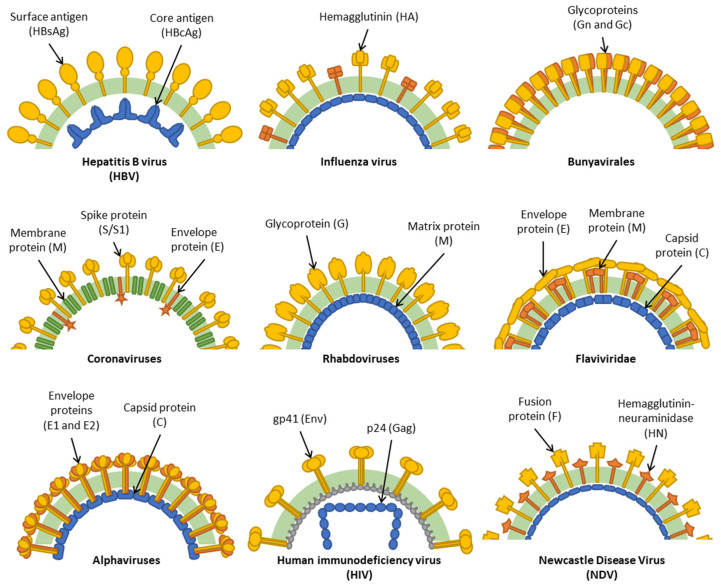
Diagrams depicting the major structural elements of the virus clades described in this review. Background dark green band represents the lipid envelope, and other elements represent viral structural proteins. Blue represents capsid proteins while yellow/orange represents membrane-bound surface glycoproteins.

The first VLP of an enveloped virus and, indeed, the first VLP of any virus, to be produced in plants was comprised of the surface antigen of HBV (HBsAg; [9]) (family *Hepadnaviridae*, reverse transcribing virus with partially double-stranded circular DNA). During HBV infection, this protein interacts with phospholipids from the host cell membranes to form both viral and subviral particles (Figure 1). Despite only low levels of HBsAg accumulating in the leaves of transgenic tobacco plants (0.01% of soluble leaf protein), it was found to assemble into structures resembling subviral particles in terms of size, buoyant density and antigenicity. Subsequently, the immunogenicity of the tobacco-derived HBsAg was demonstrated after parenteral administration to experimental mice [16]. HBsAg has been produced in various edible plants including lupin, potato, banana, tomato and lettuce, and is typically effective at eliciting an immune response against HBV when supplied orally to mice and humans [17,18,19,20,21,22,23]. More detailed structural analysis also revealed that HBsAg produced in transgenic potato tubers and in two cell lines in culture was extensively cross-linked by disulphide bonds and formed tubular structures [24], while Kong et al. [19] showed that HBsAg appeared to form particles accumulating in membrane-bound vesicles in transgenic potato. Later, Huang et al. [25] demonstrated that HBsAg forms VLPs when transiently overexpressed in *Nicotiana benthamiana*. Such transient methods were subsequently used to produce increased levels of HBsAg in plants [26,27], which makes HBsAg one of the great success stories of molecular farming of enveloped viruses from a biochemistry perspective. From an industrial perspective however, it is noteworthy that there does not appear to have been a clinical development of plant-produced HBsAg vaccine candidates since the first two landmark phase 1 clinical trials [18,20]. This points to unresolved issues of production scaling and cost in comparison to more established yeast-based expression [28,29], along with perhaps undue hopes being placed in oral formulations with minimal processing of plant tissue (which introduces numerous issues such as batch-to-batch reproducibility and the difficulty of defining an antigen dose).

In addition to HBsAg, particles of the HBV core antigen, HBcAg, have been produced in plants, initially through stable transgenesis but subsequently mainly via transient expression [27,30,31,32]. Although such particles can potentially form the basis of vaccines against HBV [33], they have mainly been deployed as a method of presenting foreign antigenic sequences, including those from enveloped viruses such as dengue virus [34,35,36]. These applications are discussed in the section dealing with flaviviruses (Section 8 below).

## 4. Influenza Viruses

Most of the work on plant-produced antigens of influenza virus (a segmented negative-sense RNA virus, family *Orthomyxoviridae*), and VLPs thereof, came after the initial successes with HBsAg. Antigen display of one of the major surface glycoproteins, haemagglutinin (HA, see Figure 1), or epitopes thereof, either on the surface of CPMV [37], HBcAg [38], TMV [39], nanodiamond particles [40] or flagellin [41], often stimulates a solid immune response in animals, but they have not been tested in human trials to date. In contrast, a large body of work has described the expression and purification of HA from a variety of serotypes without necessarily attempting to specifically form VLPs, enveloped or otherwise. In some cases, HA has been produced as a fusion to a carrier or oligomerization scaffold to aid immunogenicity [42,43,44,45,46,47,48]. In these and other studies, protein accumulation was improved by the removal of the HA transmembrane domain (TM) and/or ER retention without compromising immunogenicity or efficacy in animal models [48,49,50,51,52,53]. While expression has mainly been achieved through transient expression in *Nicotiana* leaves, expression of immunogenic HA in tobacco seeds was also shown to be possible [54], as was the expression of influenza nucleoprotein in maize seeds [55]. The numerous successes in animal models supported the clinical development of some of the subunit HA candidates described above, with two phase 1 trials reported [56,57]. Unfortunately, the results of these provide a good example of pre-clinical data being poor predictors of clinical success: while both trials demonstrated safety and tolerability, immunogenicity of the plant-based monomeric HA candidates in both of these trials was underwhelming (for reasons that are unclear) and has not, so far, led to further clinical development.

To some extent, much of the important work described above has tended to be eclipsed by the industrial and clinical development of influenza VLPs by the Canadian company, Medicago. The seminal paper by D’Aoust et al. [10] described the production of HA-only VLPs from influenza serotype H5N1 via transient expression in *Nicotiana benthamiana*. This paper demonstrated not only VLP formation, but also protective immunity in a mouse model at low doses. From that point onwards, clinical as well as industrial development of HA VLPs went from strength to strength, initially with monovalent VLP vaccine candidates against pandemic strains such as H5N1 [58,59] and H7N9 [60]. This was quickly followed with a quadrivalent HA VLP formulation for seasonal flu, which successfully completed phase 1 [61], phase 2 [62] and phase 3 clinical trials [63]. Overall, these studies demonstrate good tolerability, safety and immunogenicity. As for efficacy, the two phase 3 trials described in Ward et al. [63] demonstrated that plant-produced HA VLPs provide significant protection against respiratory illness and influenza-like illness caused by influenza viruses in adults of all ages, in a manner that is comparable to commercial egg-based seasonal flu vaccines. This vaccine is currently under review by public health authorities in various countries. It is important to note that while Medicago is undoubtedly the world leader in plant-produced influenza VLPs, they are not the only group to have demonstrated HA VLP production in plants. For example, Rybicki [11] demonstrated H5N1 HA VLP extraction from apoplastic washes of *N. benthamiana* leaves, and more recently Smith et al. [64] demonstrated the potential of plant-produced H6N2 HA as a veterinary vaccine for poultry.

## 5. Bunyavirales

The success of influenza VLP production in plants has had a knock-on effect on the production of VLPs of the *Bunyavirales* order (segmented negative-sense RNA viruses): recent work carried out in South Africa led to the production of rift valley fever virus (RVFV) VLPs in *N. benthamiana* via transient expression [65]. Notably, the transmembrane domain of the RVFV Gn protein (Figure 1) was replaced with that of influenza HA from an H5N1 strain, which allowed VLPs to be produced and purified, and these were shown to elicit a specific antibody response in mice [65]. Previously, the Gn/Gc glycoprotein of Crimean-Congo hemorrhagic fever virus (CCHFV) was expressed in transgenic tobacco, and feeding the leaves and roots to mice elicited specific IgA and IgG production; however, no attempt to purify or demonstrate VLP assembly was made [66]. Similarly, the N protein and a truncated version of Gn were produced in transgenic *Arabidopsis* and the proteins were shown to be immunogenic when the plants were fed to mice [67].

## 6. Coronaviruses

The ongoing COVID-19 pandemic, caused by SARS-CoV-2 (non-segmented positive-sense RNA, family *Coronaviridae*), has mobilised the scientific community, and the plant molecular farming community is no exception [68]. The American company iBio has announced that it is developing a plant-produced subunit vaccine candidate based on segments of SARS-CoV-2 spike (S) major surface glycoprotein (Figure 1) fused to LickM, a carrier protein derived from *Clostridium thermocellum* b-1,3-1,4-glucanase which has previously been used with influenza HA [42]. Similarly, Kentucky BioProcessing has also announced that it is developing a plant-produced subunit vaccine candidate, which is currently being tested in a phase 1–2 clinical trial (NCT04473690). No scientific data on either of these candidate vaccines has yet been published; however, there have been pre-published reports by a separate group of the transient expression of S1 (the surface-exposed region of the S glycoprotein) and nucleocapsid (N) proteins in plants for potential use as subunit vaccine candidates [69].

The first report in the scientific literature of a plant-produced vaccine candidate against SARS-CoV-2 is from Medicago, who managed to use their experience with influenza VLPs (see above) to produce a VLP vaccine candidate against the new coronavirus threat. This VLP consists of the SARS-CoV-2 S protein with a number of modifications: a few stabilising point mutations, a plant signal peptide instead of the native sequence, and the transmembrane domain and cytoplasmic tail of S were replaced with the equivalent sequences from influenza H5 HA [70], a strategy reminiscent of that used for RVFV (see above). This VLP was tested in humans in a phase 1 trial which showed very favourable safety and tolerability as well as promising immunogenicity. A phase 2–3 clinical trial with this VLP is ongoing (NCT04636697).

Before the SARS-CoV-2 pandemic, there were several attempts at producing vaccine candidates against several coronaviruses in plants. Li et al. [71] expressed the S1 protein (the ectodomain region of S which includes the receptor-binding domain) of SARS-CoV transiently in tobacco leaves as well as stably in transgenic tobacco and lettuce and transplastomic tobacco, although no immunogenicity experiments were performed. Other groups expressed the N [72] or M [73] proteins of SARS-CoV in *N. benthamiana* and demonstrated basic immunogenicity. There were also numerous examples with veterinary coronaviruses, such as the production of stable transgenic lines of *Arabidopsis* [74] and potato [75] expressing the S protein of the pig-infecting virus, transmissible gastroenteritis coronavirus (TGEV). The expressed proteins were found to stimulate a specific antibody response in mice. There have also been efforts to develop plant-based vaccines against porcine epidemic diarrhoea virus (PEDV), a major cause of losses in agriculture. Early work was based on the expression of a synthetic neutralising epitope of this virus (termed COE), either fused to cholera toxin subunit B (CTB) and expressed in lettuce [76,77] or fused to M-cell targeting ligand Co1 and expressed in rice calli [78]. Later work involved the expression of an S protein epitope fused to CTB either transiently in *N. benthamiana* [79] or transplastomically in *Nicotiana tabacum* [80]. More recently, the expression of the COE synthetic epitope fused to a trimerization motif (C-terminal isoleucine zipper trimerization motif GCN4pII) has been expressed transiently in *N. benthamiana* and was shown to stimulate neutralising antibody production in mice [81]. Moreover, the much larger S1 coding region was expressed in transgenic maize and was shown to elicit a neutralising antibody response in pigs after oral administration [82].

**Figure 2:**
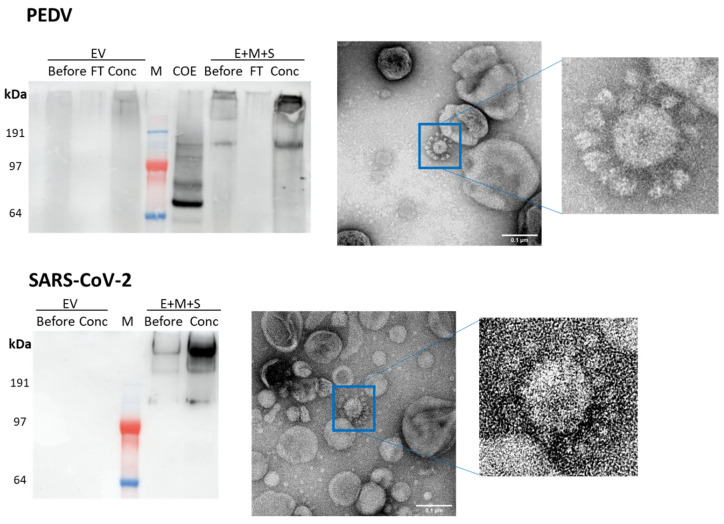
Transient co-expression of E, M and S coronavirus proteins leads to the formation of possible VLPs. Top: porcine epidemic diarrhoea virus, bottom: SARS-CoV-2. The E, M and S proteins are co-expressed in *N. benthamiana* and purified by sucrose cushion followed by a sucrose gradient then desalting column before concentration on a spin column concentrator. Left: anti-S Western blots on samples from co-expression of coronavirus proteins (E + M + S) or empty vector negative control (EV). Before: sample recovered from desalting column, FT: flow-through from spin-column concentrator, Conc: retentate from spin column concentrator, M: marker, COE: positive control. Right: transmission electron micrographs of Conc samples shown in Western blots. Samples stained with 2% (*w*/*v*) uranyl acetate, scale bars are 100 nm.

In our group, we have analysed the incorporation into VLPs of the E, M and S proteins of PEDV and SARS-CoV-2 expressed in *N. benthamiana*. Preliminary findings suggest the formation of VLPs (Figure 2), although low yield and difficulties with purification make quantification and further characterization difficult at this stage.

## 7. Rhabdoviruses

Rhabdoviruses have negative-sense ssRNA genomes contained within a canonically bullet-shaped particle [83]. These particles consist of three key structural proteins: a nucleoprotein (N-protein) coating the RNA genome, a matrix protein (M-protein) that lines the lumenal face of a host-derived lipid membrane, and a glycoprotein (G-protein) that spans this membrane and protrudes from the surface [84] (Figure 1). As one of the relatively few families of enveloped viruses naturally infecting plants, Rhabdoviruses theoretically present an interesting opportunity for cross-pollination between animal and plant virology. However, the reality so far has been that plant rhabdoviruses have not proven to be of much help in aiding the production of animal/human rhabdovirus VLPs in plants, mostly because plant rhabdoviruses themselves are notoriously low-yielding and difficult to work with [85,86,87]. In fact, there is even an example of a plant rhabdovirus G-protein ectodomain being produced in mammalian cell cultures to generate rabbit antiserum to study this virus in plants [88].

The surface-exposed G protein is a major Rhabdovirus antigen, and alongside its role in cell entry [84] is the key target for neutralising antibodies [89]. As such, many, but not all [90,91] approaches to generating novel Rhabdovirus vaccines in plants have focused on the production and presentation of rabies G protein to prime effective immune responses. Some of the earliest investigations into plant-derived Rhabdovirus vaccine candidates used recombinant plant viruses to display antigenic sequences [91,92]. Initially, a key concern with this approach was that without additional targeting sequences [93], the displayed epitope will not undergo glycosylation, which is thought to be a key factor in rabies G protein antigenicity and folding [84,94]. However, the immunological data demonstrated that the absence of glycosylation did not prevent the induction of IgG [91] or IgA [92] responses in mice or humans, and importantly, reduced mortality in animal challenge experiments done in those studies.

Having established that plant-specific modifications are not detrimental to Rhabdovirus vaccine candidates, an obvious question arises regarding plant-based expression systems—can plants produce effective edible vaccines against rabies infection? Oral vaccination has shown great success in reducing rabies in wild reservoirs; targeting canids in central and eastern Europe with recombinant or attenuated virus encased in bait led to the almost complete elimination of rabies from wild populations across central Europe over a 20-year period [95]. Herbivorous livestock can act as important spill-over hosts for rabies infection, and are therefore prime targets for an effective plant-based oral vaccine that can be easily introduced into their diet.

Alongside attaining reasonable yields in the commonly used tobacco species (*Nicotiana benthamiana* and *N. tabacum*), research has shown that several crop species can generate sufficient Rhabdovirus antigens in a reasonable biomass to make introduction of the vaccine via feeding viable without significant processing, with reports of 1% total soluble protein, or 20–35 µg G-protein per gram fresh weight. These edible vaccine candidates focused on crops amenable to genetic transformation: spinach [91,92], maize [96], and tomato, for which several tissues have shown reasonable antigen yields [90,97,98]. Oral vaccination using these three crops showed mixed results, however. Although injection of tomato extract expressing N-protein decreased mortality by 50% in mice after lethal challenge [90], oral vaccination did not provide protection, despite priming an immune response. More promising results were seen using transgenic spinach as a feedstock. In this instance, oral vaccination with recombinant alfalfa mosaic virus (AlMV) displaying a combination of N- and G-protein epitopes improved weight gain in mice following challenge with an attenuated virus strain [92]. In addition to the additive effect of N- and G-protein epitopes [99], the potential for intrinsic differences between the two expression systems, or improved gastric stability and immunogenicity of the antigen fused to the AlMV carrier, is unclear. More recently, maize has been engineered to produce an oral rabies virus vaccine [96]. Maize seeds are an attractive platform for Rhabdovirus oral vaccine production given their relatively high accumulation of protein, and the ease of long-term storage under ambient conditions [100]. In this instance, transgenic maize lines were generated showing accumulation of a soluble form of rabies G-protein, which, when fed to sheep, not only induced neutralizing antibody production, but also gave dose-dependent protection against lethal challenge [96].

**Figure 3:**
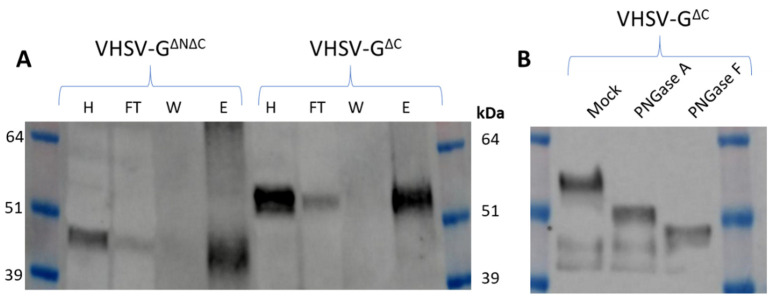
VHSV G protein produced in plants is glycosylated. His-tagged N- and C-terminally truncated G protein (VHSV-G^ΔNΔC^) and C-terminally truncated G protein (VHSV-G^ΔC^) were transiently expressed in *N. benthamiana*. The N-terminal truncation removes the native secretion signal and hydrophobic region, while the C-terminal truncation removes the transmembrane domain. (**A**) Anti-G Western blot of homogenate (H), flow-through (FT), wash (W) and elution (E) fractions of nickel column affinity purification for both constructs. (**B**) anti-G Western blot of affinity-purified VHSV-G^ΔC^ after mock deglycosylation treatment or treatment with PNGase A and F. The band shift is indicative of glycosylation.

Although much of the focus for plant-derived rabies vaccines has centred on terrestrial infection, Rhabdoviruses are also an important infectious agent in marine environments [101]. With aquaculture set to play an important role in food security in coming decades, better methods to protect stock health are needed. Edible vaccines have been touted as an important technology for aquaculture [102], and work in our group shows that *N. benthamiana* is able to accumulate and glycosylate soluble variants of a G-protein from a marine rhabdovirus, viral haemorrhagic septicaemia virus (VHSV, Figure 3), although enveloped VLPs were never observed. Regarding the glycosylation, the plant-derived G-protein we present shows a more consistent and potentially a more extensive glycosylation than insect- or insect-cell derived counterparts [103,104], as indicated by a mass shift of around 10 kDa following treatment with deglycosylating enzymes. This plant-derived glycosylation appears comparable in its extent to G-protein produced using traditional fish cell culture [105,106]; however, due to the differences in glycosylation performed between plant and animal cells, the composition of glycans is likely to be significantly different. Nevertheless, insect-cell produced G-protein (despite being less glycosylated and more heterogenous than native) still protects trout against VHSV challenge [103], and *E. coli*—produced, non-glycosylated G protein (refolded from inclusion bodies) could induce neutralizing antibodies in trout [107], indicating that perfect recapitulation of native glycosylation is not essential for vaccine candidates. Other groups have demonstrated that regions of the VHSV G-protein attached to the carrier molecule CTB can also be expressed in *N. benthamiana* [108], though immunogenicity was not studied. Moving away from tobacco species, with their high concentrations of defensive secondary metabolites, into more suitable plant species for fish feeding will be essential for this technology to advance.

Despite the fact that this virus family comprises both animal- and plant-infecting viruses, there are no published examples to date of plant-produced enveloped rhabdovirus VLPs. There is, however, a patent that describes a method of doing just this for rabies VLPs, involving the transient co-expression of M and G proteins in *N. benthamiana* followed by plant cell wall lysis as the basis for VLP extraction [109]. Perhaps unsurprisingly, this patent was filed by Medicago, but no scientific publication has accompanied this patent or shown microscopic evidence of these plant-made rabies VLPs. Press releases in 2012 suggested imminent clinical trials, though we could not find reports of the results of these trials, or even signs that such trials took place. At the time of writing, there is no mention of a rabies vaccine candidate on the Medicago website. Regardless, this patent and the studies mentioned demonstrate that vaccination with plant-derived rabies vaccines (subunit or VLP) by either injection or feeding can provide protection against infection with an authentic virus (at least in non-human animals), highlighting the potential of this technology as a tool in rhabdovirus vaccination efforts, particularly in agriculture and aquaculture.

## 8. Flaviviridae

Most of the earlier work on developing vaccine candidates against *Flaviviridae* (non-segmented positive-strand RNA viruses) in plants have focused on hepatitis C virus (HCV), with limited success. There are numerous examples of expression of an immunogenic epitope from the E2 surface glycoprotein (Figure 1), the R9 mimotope, being fused to a carrier, the first example being CTB [110]. Later, this mimotope was fused to the surface of AlMV [111], HBsAg [112], and CMV [113,114,115], in an attempt to develop oral vaccines using edible crop plants as expression hosts [116]. It was later shown that the entire E1 and E2 major surface glycoproteins of HCV could be expressed in lettuce, and this lettuce was shown to be capable of behaving as an oral boost in mice that had been previously primed with mammalian cell-produced E1E2 [117].

**Figure 4:**
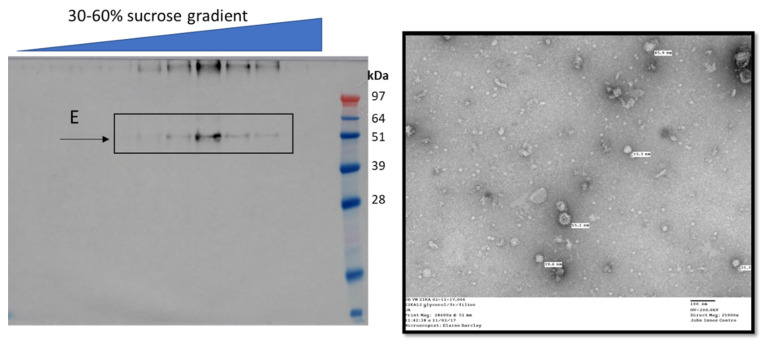
ZIKV prM-E expression in plants yields possible VLPs. The prM-E region of ZIKV was transiently expressed in *N. benthamiana* and purified on a sucrose cushion followed by desalting and concentration on a spin filter concentrator. The concentrate was then further purified over a 30–60% (*w*/*v*) sucrose gradient and the fractions were assayed by anti-E Western blot (**left**). The positive fractions (indicated) were pooled, buffer-exchanged and concentrated on a spin filter concentrator and imaged by transmission electron microscopy (**right**), revealing possible VLPs 35–55 nm in size. Samples stained with 2% (*w*/*v*) uranyl acetate, scale bar is 100 nm.

There have also been attempts at producing antigens from Japanese encephalitis virus (JEV), notably the E surface glycoprotein in transgenic rice, which was shown to elicit an immune response after oral or intraperitoneal administration into mice [118]. The domain III of the E protein displayed on the surface of the potexvirus bamboo mosaic virus, was found to stimulate neutralising antibody production in mice [119]. There has also been a considerable amount of work on developing plant-produced West Nile virus (WNV) vaccine candidates, extensively reviewed by Chen [120]. This review describes work involving production in plants of domain III of the E protein of WNV (wEDIII), which was shown to be immunogenic [121] and probably even protective in mice [122], and hints at the production of the entire E protein as well as WNV VLPs produced in plants from expression of a prM-E construct, though this does not appear to have been published. There are, however, examples of wEDIII fused to HBcAg, which yields immunogenic core-like particles that stimulate an immune response against WNV in mice [123]. Using a similar strategy, domain III of the E protein of Zika virus (ZIKV, zEDIII) was displayed on the surface of HBcAg particles, and these were found to be highly immunogenic in mice [124]. In a later study, these HBcAg-based zEDIII VLPs were found to behave synergistically when co-administered to mice alongside recombinant immune complexes (RIC) consisting of plant-produced zEDIII fused to anti-ZIKV antibodies [125]. The entire ectodomain of ZIKV E was also produced in plants and were found to be immunogenic in mice, though no attempt was made to produce VLPs [126]. Work in our group has shown that expressing a ZIKV prM-E construct transiently in *N. benthamiana* leads to the accumulation of E protein in a dense form that migrates into a sucrose gradient, but low yield and difficulties with purification and concentration never allowed us to definitively confirm VLP formation (Figure 4).

The RIC technology mentioned above had previously been used to demonstrate immunogenicity in mice of a plant-produced vaccine candidate against dengue virus (DENV), through the fusion of domain III of the E protein of DENV (dEDIII) to a chimeric antibody construct [127]. This is just one example out of many in which dEDIII has been produced in plants: it had already been produced through both transient expression in *N. benthamiana* [128] and stable transformation of *N. tabacum* [129]. Martínez et al. [130] showed that a much larger (though not full-length) version of DENV E could be produced by transient expression in *N. benthamiana*, and that dEDIII could be displayed on the surface of HBcAg core-like particles, a strategy which was later confirmed by Pang et al. [35] to produce a specific immune response in mice. The production of dEDIII in the chloroplasts of tobacco [131] and lettuce [132] has also been demonstrated, in early attempts to develop oral vaccine options. Moreover, a prM-E construct of DENV serotype 3 was used to transform tobacco chloroplasts, with some evidence of VLP formation [133]. Finally, recent work in our group has shown that transient co-expression of a DENV serotype 1 structural protein construct with a DENV 1 non-structural protein construct in *N. benthamiana* results in the formation of prM-E VLPs, which are immunogenic in mice [134]. Interestingly, this strategy could not be replicated for the other serotypes of DENV. We also demonstrated that dEDIII could be fused to the inside of bluetongue virus core-like particles, but these did not appear to be very immunogenic.

## 9. Alphaviruses

**Figure 5:**
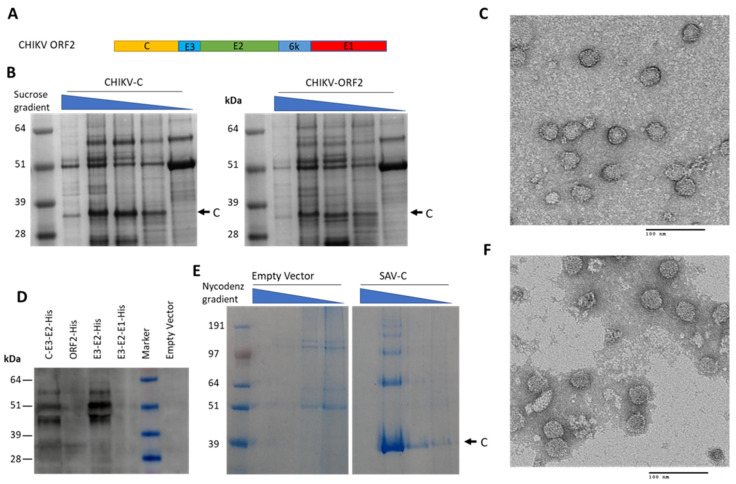
Alphavirus structural proteins are expressed in plants and self-assemble into capsid-like structures. Proteins of CHIKV (**A**–**D**) and SAV (**E**,**F**) were transiently expressed in *N. benthamiana* from pEAQ-HT constructs for capsid protein, the entire open reading frame 2 (ORF2), or portions of ORF2 with C-terminal histidine tag. (**A**) Schematic representation of CHIKV-ORF2. (**B**) InstantBlue-stained SDS-PAGE gels of gradient fractions from CHIKV-C and CHIKV-ORF2-expressing tissue. CHIKV capsid protein band indicated. (**C**) Transmission electron micrograph of CHIKV-C sucrose gradient fraction. (**D**) Western blot of crude leaf extract probed with anti-his antibody, showing production of his-tagged CHIKV-E2. (**E**) InstantBlue-stained SDS-PAGE gel of Nycodenz gradient fractions for purification of SAV capsid protein (N-terminally his-tagged) or empty vector control. SAV capsid protein band indicated. (**F**) Transmission electron micrograph of N-terminally His-tagged SAV-C Nycodenz gradient fraction. Samples stained with 2% (*w*/*v*) uranyl acetate before TEM imaging, scale bars 100 nm.

Interest in using plants as a cost-effective production platform for Alphavirus (non-segmented positive-strand RNA virus, family *Togaviridae*) vaccines arose after an outbreak of chikungunya virus (CHIKV) in the Americas in 2013–2014 [135]. CHIKV VLPs had already been successfully produced in other eukaryotic systems by expression of open reading frame 2 (ORF2, Figure 5A), which encodes all the structural proteins necessary for VLP assembly [136] (Figure 1). It was proposed that plants might be used to produce VLPs in a similar way, or to follow a strategy of antigen display on CTB [137], but no such studies have been published to date.

Our group has attempted the production of CHIKV VLPs through transient expression of ORF2 in its entirety, or shorter fragments thereof. Capsid protein (C) was efficiently produced and self-assembled into capsid-like particles when expressed alone or as part of ORF2, indicating that autoproteolytic cleavage of C occurs in plants (Figure 5B,C). However, of the envelope glycoproteins expressed from ORF2, only E2 was detected by Western blot when tagged with a C-terminal histidine-tag (Figure 5D) and no structures resembling full CHIKV VLPs were observed. Similarly, we have expressed salmonid alphavirus (SAV) capsid protein and shown capsid-like particle assembly by density gradient purification of SAV C and transmission electron microscopy (Figure 5E,F), but again we were not successful in expressing SAV envelope proteins in such a manner as to obtain enveloped VLPs. Our preliminary data and the lack of published studies strongly suggest that plants may not be a viable production platform for enveloped alphavirus VLPs, although alphavirus capsid-like particles seem relatively straightforward to produce in plants.

## 10. HIV

The examples of plant-produced vaccine candidates against human immunodeficiency virus (HIV, non-segmented RNA reverse transcribing virus, family *Retroviridae*) are too numerous to review exhaustively here, and readers are referred to previous reviews [11,138,139]. The earliest work focused on small epitopes of HIV proteins displayed on the surface of plant viruses, such as CPMV, which was used to display a 22 amino acid epitope from gp41 of HIV-1 [140]. Parenteral administration of the purified particles to mice produced sera that were neutralising against three strains of HIV-1 [141,142]. These sera contained HIV-1-specific antibodies to two distinct epitopes within the gp41 peptide, one neutralising and one non-neutralising [143].

Whole HIV proteins have been expressed in transgenic plants, the first of which was the p24 capsid protein (see Figure 1) produced in transgenic tobacco plants [144], though no immunology was carried out. The Tat protein was then expressed in spinach, and although oral administration to mice did not stimulate a specific antibody response, it was found that it did have a priming effect on subsequent boosting with a Tat-specific DNA vaccine [145]. Various small epitopes or synthetic multiepitope constructs have also been expressed in plants, notably as fusions to HBsAg in tomatoes [146], *Arabidopsis* and tobacco [147,148]. While the authors of these studies did not actually demonstrate VLP formation, they did demonstrate that oral administration of the transgenic plant tissue could stimulate an immune response in mice.

Improvements in construct design and subcellular localisation has led to improved yields of recombinant proteins [149] and Gag-derived capsid-like particle formation in transplastomic *N. tabacum* [150] as well as transgenic *N. benthamiana* [151]. In the latter example, there were strong hints that stably expressed Gag interacted with transiently co-expressed gp41 (the membrane-proximal external region of Env) to make enveloped VLPs, and these putative VLPs were found to stimulate a strong immune response against Gag in mice, though the immune response against gp41 was found to be comparatively weaker [152]. The much larger gp140 version of soluble Env has also been produced both stably and transiently in tobacco [153] as well as transiently in *N. benthamiana* [154]. In the latter case, purified recovered yields of gp140 were low (<2 mg of purified protein per kg of infiltrated leaf tissue), but there was evidence that this purified gp140 was in a trimeric form, and these trimers were immunogenic in rabbits.

## 11. Newcastle Disease Virus

This review would be incomplete without a mention of Newcastle disease virus (non-segmented negative-sense RNA virus, family *Paramyxoviridae*), which causes significant disease burden in both farmed poultry and wild bird populations [155]. The key antigenic targets for this virus are the Fusion (F) and Haemagglutinin-neuraminidase (HN) surface glycoproteins [156,157] (Figure 1). Importantly for vaccine design, the oligomeric state of the HN protein, driven in part by the presence of the transmembrane stalk, does not impact recognition by monoclonal antibodies [158,159]. Despite early work showing the viability of displaying NDV epitopes using CMV [160,161,162], most research into plant-derived NDV vaccines has focussed on expression of HN or F subunits. Various expression hosts have been explored for NDV vaccine production including potato [163], rice [164], maize [165,166], rapeseed [167], *N. benthamiana* [168], *N. tabacum* [169], and tobacco cell culture [170] with varying success. Modifications to constructs, such as the use of appropriate signal peptides and inclusion of ER retention signals, can enhance NDV antigen yield [168]; however, the highest reported accumulation of NDV antigen stands at 3% of total soluble protein in maize [165], with more typical yields reported across multiple plant species ranging from 0.18–0.8% of total soluble protein [166,167,170] and some yielding below 0.07% [169].

Immunization by injection with plant extracts can induce anti-NDV IgG [163]. The viability of an oral delivery approach was first demonstrated by feeding transgenic potato leaves to mice, inducing anti-NDV IgG and IgA responses against both HN and F proteins [163]. The positive control of mice fed non-transgenic leaves soaked in NDV showed substantial IgG responses, but not significant IgA titres, suggesting that oral vaccination with an antigen that is contained within a cellular environment (i.e., orally administered plant tissue) could have benefits over oral vaccination with purified components.

As the main target for edible NDV vaccines are farmed poultry, seed-based antigen production has great appeal given both the ease of introduction into the diet via existing feeding methods, and the long-term stability of recombinant antigens in seeds stored under ambient conditions [100]. For NDV, chicks fed transgenic rapeseed or maize seeds produce antibodies against both HN and F proteins when these are expressed as fused ectodomains [167] or by co-expression of subunits via a single plasmid [166]. However, antibody responses tended to be significantly slower and less robust than those stimulated by existing commercial vaccines. Seed-based oral vaccination against NDV remains a viable approach, however, as demonstrated by Guerrero-Andrade et al. [165], where chicks fed dough from transgenic maize expressing F protein showed 100% survival against lethal challenge with NDV.

Despite the apparent promise of oral vaccine, the major milestone regarding NDV in the history of plant-produced vaccine candidates is a purified formulation for injection: In 2006, a veterinary vaccine based on HN produced in a *N. benthamiana* cell culture system became the first ever plant-produced vaccine to be licensed for commercial use to protect chickens from this virus [171,172]. However, the owners of this technology, Dow AgroSciences, never actually commercialised it, presumably for economic reasons.

## 12. Other Virus Families

This review has focused on the virus families for which there has been the most progress in terms of making antigens through plant molecular farming methods. There are, however, examples from other families of enveloped viruses which deserve mention. For example, a synthetic multiepitope peptide from ebolavirus (*Filoviridae*) surface glycoprotein (GP) was expressed in transgenic tobacco [173], and the GP1 protein was expressed in *N. benthamiana* as part of an immune complex strategy (similar to what has been described above for flaviviruses) and was found to be protective in a mouse lethal challenge model [174,175]. There have also been attempts at making vaccine candidates against human respiratory syncytial virus (*Pneumoviridae*) by fusing small epitopes of the G protein to alfalfa mosaic virus coat protein [176,177]. Furthermore, vaccine candidates against measles virus (*Paramyxoviridae*) have been expressed in plants: a polyepitope sequence was expressed in transgenic carrot [178], and full-length hemagglutinin (H) of measles virus was produced in transgenic tobacco [179,180], carrot [181], and lettuce [182], all of which were immunogenic in mice [183].

## 13. Concluding Remarks

The past 30 years have seen the development of plant-produced vaccine candidates against enveloped viruses of both medical and veterinary concern. The main challenges have included low expression of antigen, concerns about plant glycosylation patterns, and difficulties with extraction and purification. The first issue has been addressed through a combination of improvements in plant transient expression systems [184,185] and protein engineering strategies such as optimising subcellular localisation and transmembrane domains [50,52] or expressing carrier proteins [42,45] or chaperones [186,187]. Plant glycosylation, meanwhile, does not appear to constitute a significant stumbling block for plant-produced vaccines, despite initial concerns. Plant glycans do not generally compromise specificity of the immune response, and developments in plant genome engineering are paving the way for “humanised” glycosylation patterns [138,187]. Difficulties with extraction and purification are, to a large extent, the biggest stumbling block that remains for translation of vaccine candidates from plant molecular farming research to clinical development [188,189]. In this regard, it is noteworthy that using antigen-producing plant material as oral vaccines has clearly never progressed beyond the point of ambition, at least for human vaccine candidates. Indeed, regulatory and industrial realities surrounding dosage control as well as good manufacturing practice [190], have all but killed the idea of edible vaccines that would not require expensive and time-consuming purification or formulation (although this may not apply to certain veterinary applications). It is striking that the HBV VLP vaccine candidate trials, which consisted of feeding human volunteers HBsAg-producing lettuce [18] or uncooked potato [20], never progressed beyond early phase clinical testing. In contrast, the influenza [63] and SARS-CoV-2 [70] VLPs which are currently at late-stage clinical development are both purified products formulated for injection.

While examples of plant-produced enveloped virus vaccine candidates can be found from a wide variety of enveloped virus families, the only examples of products clinically tested in humans to date are hepatitis B virus [18,20], influenza virus [56,57,58,59,60,61,62,63], and SARS-CoV-2 [70]. There have, however, been numerous pre-clinical trials in animal models, and some trials of potential veterinary vaccines in target animals [54,64,102,165,191]. While examples of plant-produced enveloped VLPs are comparatively rare compared to non-VLP subunits or non-enveloped VLPs, they are clearly over-represented in the clinical trials carried out in humans. Indeed, the only non-VLP vaccine candidates which have been used in clinical trials are monomeric influenza HA [56,57], and the results of those trials were underwhelming compared to their previous success in pre-clinical studies. In contrast, VLPs of influenza virus and SARS-CoV-2 are still progressing in clinical development, and their licensure seems likely in the coming year. Plant-produced HBsAg VLPs, however, do not appear to have progressed in clinical development beyond phase 1. This suggests that VLPs tend to make the most promising vaccine candidates against enveloped viruses, but it could also be an indication that clinical development requires development by a well-funded private company such as Medicago, which is responsible for clinical development of both the influenza and SARS-CoV-2 VLPs. This is revealing of a gap between the academic research groups who are motivated by pushing the boundaries of the possible in plant molecular farming, and private companies which must contend with the practicalities of extraction and purification at scale, as well as regulatory imperatives. In this regard, long-term collaborations between the established academic base of plant molecular farming and the growing industry will be crucial to pushing plant-produced vaccines through the clinical pipeline: first by making the impossible, difficult; then by making the difficult, competitive.

## Data Availability

The unedited images of the western blots shown in the figures are available in the supplementary information.

## Supplementary Materials

The following are available online at https://www.mdpi.com/article/10.3390/vaccines9070780/s1.
