# Supplementary material for: Producing Vaccines against Enveloped Viruses in Plants: Making the Impossible, Difficult
Source: Vaccines (Basel). 2021 Jul 13;9(7):780. doi: 10.3390/vaccines9070780 (PMC8310165; doi:10.3390/vaccines9070780)

**Supplementary Information**

# Producing vaccines against enveloped viruses in plants: making the impossible, difficult.

Hadrien Peyret, John F.C. Steele*, Jae-Wan Jung, Eva C. Thuenemann, Yulia Meshcheriakova, George P. Lomonossoff

Department of Biochemistry and Metabolism, John Innes Centre, Norwich, UK

* Current address: Piramal Healthcare UK Ltd., Piramal Pharma Solutions, Earls Road, Grangemouth, Stirlingshire, Scotland, UK, FK3 8XG.

**Figure 2 complete western blots**

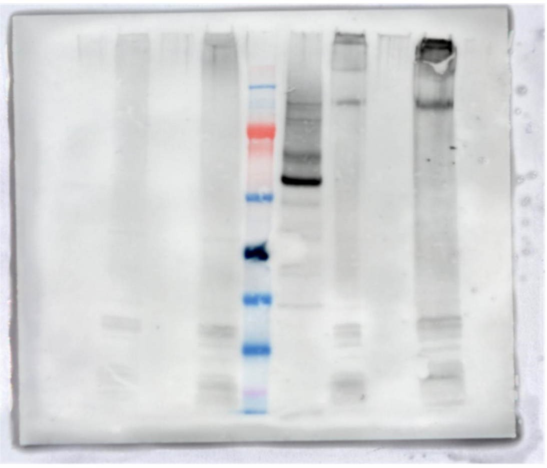

PEDV

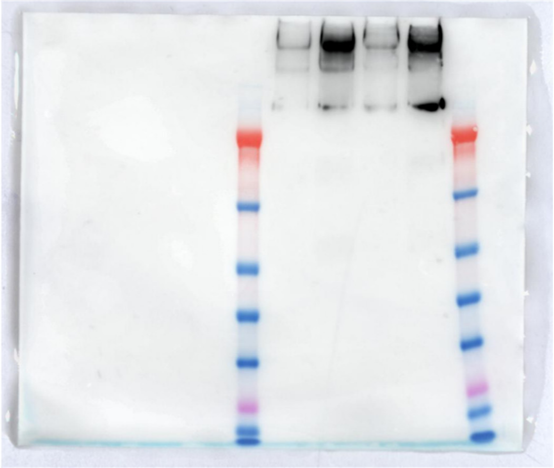

SARS-CoV-2

**Figure 3 complete western blots**

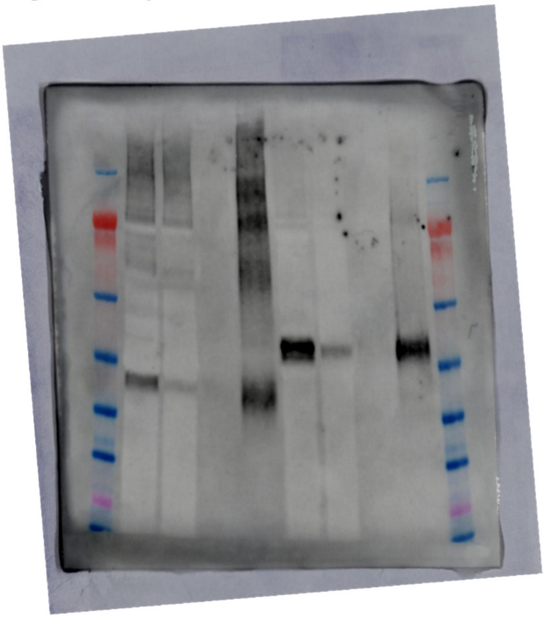

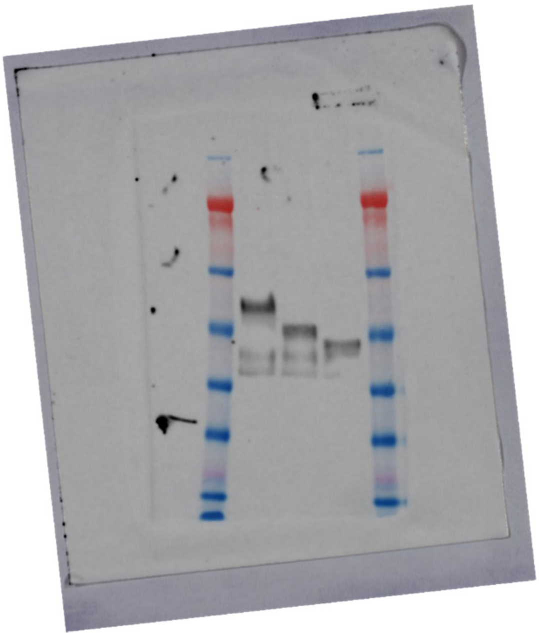

**Figure 4 complete western blot**

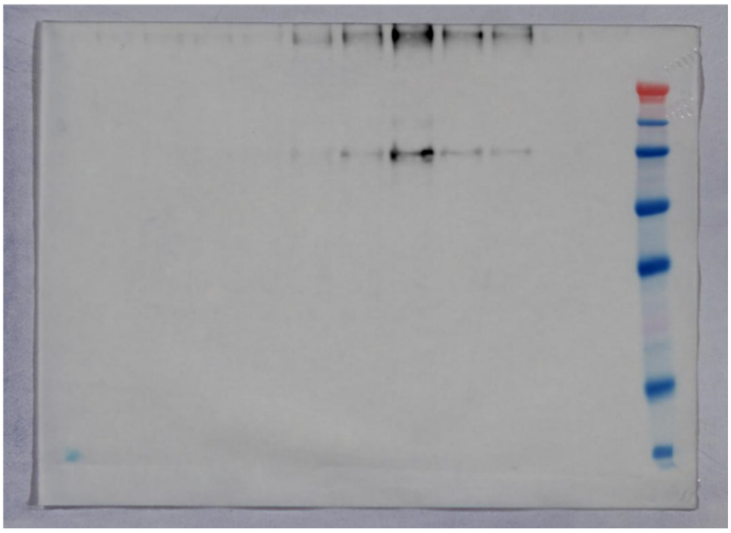

**Figure 5D complete western blot**

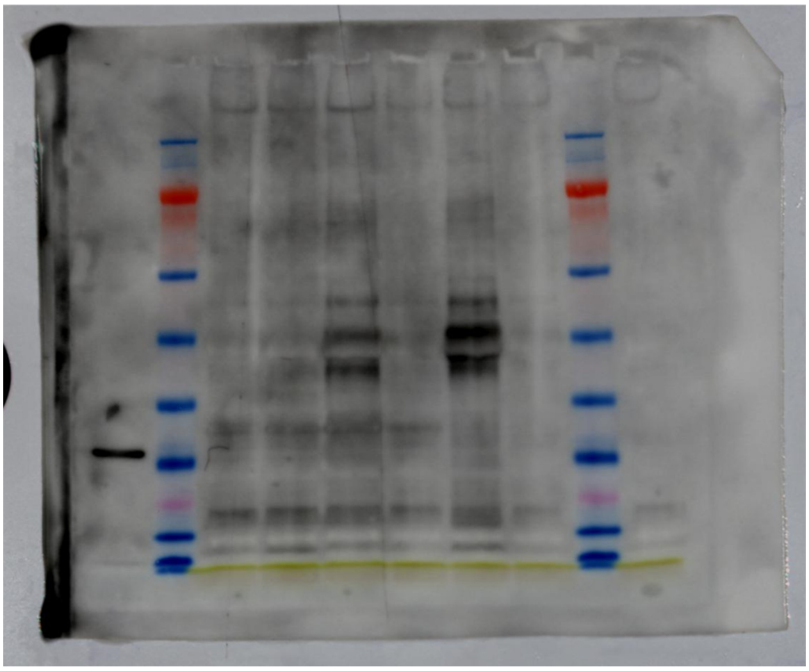

Supplement: Supplementary file 1 [file vaccines-09-00780-s001.zip › vaccines-1270626-supplementary.pdf]
